# Evaluation of Laser Powder Bed Fusion-Fabricated 316L/CuCrZr Bimetal Joint

**DOI:** 10.3390/ma18071434

**Published:** 2025-03-24

**Authors:** Wengang Zhai, Guanchun Li, Wei Zhou

**Affiliations:** 1 Singapore Centre for 3D Printing, Nanyang Technological University, 50 Nanyang Avenue, Singapore 639798, Singapore; wengang001@e.ntu.edu.sg (W.Z.); ligu0009@e.ntu.edu.sg (G.L.); 2 School of Mechanical and Aerospace Engineering, Nanyang Technological University, 50 Nanyang Avenue, Singapore 639798, Singapore

**Keywords:** additive manufacturing, dissimilar welding, multimaterial, stainless steel, CuCrZr alloy

## Abstract

While a tensile test revealing joint fracture at the base material may indicate good joint quality under certain circumstances, this conclusion might overlook the importance of examining the joint interface because exceptions can occur when one side is significantly softer. This study investigates the fabrication of a 316L/CuCrZr bimetal structure using the laser powder bed fusion (LPBF) process. Cracks were observed at the joint interface. The microhardness measured approximately 200 HV at the cracked interface and 100 HV on the CuCrZr side. Tensile testing showed that fractures occurred on the CuCrZr side, despite the presence of cracks at the bonding interface of the 316L/CuCrZr bimetal joint. Spheroids of 316L and Cu were found at the interface due to the Fe-Cu immiscibility system. This immiscibility was the main reason for the formation of cracks. This highlights the need for a thorough microstructural examination of the bonding to ensure a comprehensive quality assessment. The LPBF-fabricated 316L/CuCrZr bimetal joint exhibits a yield strength of 203.0 MPa, a UTS of 287.5 MPa, and an elongation of 15.3%.

## 1. Introduction

Multimaterial structures and functionally graded materials (FGMs) offer significant design flexibility and potential for various engineering applications [1,2]. These structures combine multiple materials, each contributing its strengths, to achieve enhanced overall performance.

Traditionally, multimaterial structures can be fabricated through various welding processes, such as arc welding [3], laser welding [4], friction stir welding [5], and brazing [6]. However, these methods are generally limited to joining materials with simple geometries. Fabricating multimaterial structures with complex geometries remains a significant challenge. The demand for single-process multimaterial fabrication is growing, and additive manufacturing provides superior control over material properties compared to conventional methods, enabling greater design freedom, improved functionality, and enhanced mechanical properties, particularly for aerospace, marine, and medical applications [7].

Fabricating multimaterial structures using additive manufacturing involves not only printing individual alloys but also joining them. It is relatively easy to join materials with similar thermal expansion coefficients, like 316L/ER70S-6 [1], 316L/Hastelloy X [8], and 316L/Inconel 718 [9]. However, joining materials with large mismatches in thermal expansion and physical properties poses significant challenges, often leading to porosity and cracks in these systems, such as 316L/CuSn10 [10], M300/Cu [11], Ti6Al4V/Cu10Sn [12], W/Cu [13,14], and 316L/K220 [15]. Sun et al. [15] studied the LPBF of a 316L stainless steel and K220 copper bimetal structure. Vertical and horizontal interfaces were fabricated and investigated. Cracks along both interfaces were observed. The mechanical properties were not studied in this work. Li et al. [16] fabricated a 316L/CuCrZr bimetal structure with an Inconel 718 interlayer. They deposited 10 layers of Inconel 718 on a 316L substrate, and then, CuCrZr alloy was deposited on the Inconel 718. Good metallurgical bonding was achieved in the bimetal joint.

Most studies on additively manufactured multimaterial structures focus on printability, microstructural evolution, and mechanical properties. However, studies that specifically evaluate dissimilar joints from a joining perspective remain limited, particularly when the materials involved exhibit significant differences in physical and mechanical properties. In this work, a 316L/CuCrZr bimetal structure was fabricated using the LPBF process. The integrity of the dissimilar joint was initially assessed through tensile testing, where fractures occurred outside the joint interface, suggesting good bonding. Cracks were observed at the interface; however, the bimetal joint did not fracture at these locations. Instead, the fracture occurred on the base material side. This finding suggests that fracture on the base material side during a tensile test is not sufficient to confirm the quality of a welded joint. A detailed microstructural examination of the weld seam is essential for a comprehensive assessment. Additionally, the formation and underlying mechanisms of the observed cracks were analyzed.

## 2. Materials and Methods

Commercial gas-atomized 316L powders and CuCrZr powders were used as the powder stock. Their nominal chemical compositions are shown in Table 1. The powder size range is 15–50 µm, and the average sizes are about 40 µm for both types of powder.

**Figure 1:**
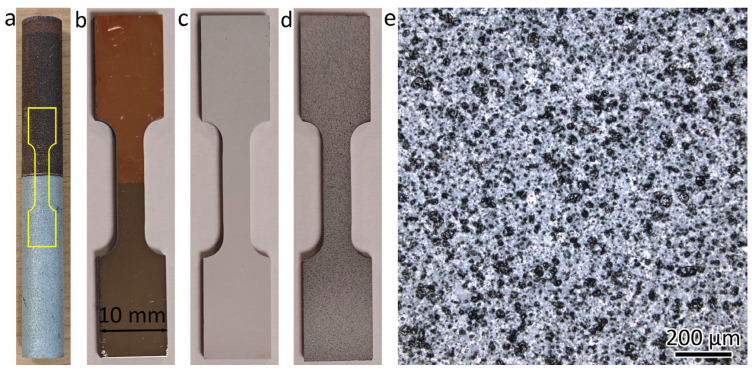
Photos showing the sample preparation for DIC test for 316L/CuCrZr structure. (**a**) LPBF-fabricated bar. (**b**) Tensile sample after grinding. (**c**) Tensile sample after white painting. (**d**) Tensile sample after black spray. (**e**) Micron-sized black spray on the tensile sample.

A commercial LPBF machine (JHL 500, Ji Hua Laboratory, Foshan, China) was used for the printing of the bimetal structure. The machine was equipped with a laser with a wavelength of 1070 nm. The laser spot size was about 100 µm at the focal point. During printing, 316L was first printed on a 316L substrate. After the printing of 316L was finished, the 316L powders were removed, and then the powder chamber and printing chamber were cleaned before loading CuCrZr powders. The machine was then re-calibrated for the printing of CuCrZr. Pure argon gas was used as the shielding gas. During printing, the oxygen level in the chambers was controlled to remain below 1000 ppm. The laser scanning direction was rotated 90˚ between two adjacent layers. The detailed printing parameters for 316L and CuCrZr are listed in Table 2. The LPBF-fabricated 316L/CuCrZr bimetal structure had a diameter of 10 mm and a total height of 100 mm. The height was 50 mm for 316L and 50 mm for CuCrZr. A photo of the 316L/CuCrZr bimetal structure is shown in Figure 1a.

The samples for microstructural analysis were ground using SiC sandpapers (320–2000 grit). After grinding, a 40 nm silica oxide polishing suspension (Struers, Copenhagen, Denmark was used for polishing. The microstructures were observed using an Olympus BX53M optical microscope (Tokyo, Japan) equipped with an Olympus DP23 CCD camera (Tokyo, Japan), a scanning electron microscope (JEOL JSM-7600F, Tokyo, Japan), and electron backscattered diffraction (EBSD, Oxford Instruments, Abingdon, UK). An accelerating voltage of 10 kV was used for SEM observations. A step size of 0.8 μm and an accelerating voltage of 20 kV were used for EBSD measurements.

The microhardness tests were conducted using a Galileo Durometria Hardness Tester (ISOSCAN HV2, Antegnate, Italy) with a load of 300 g and a dwell time of 15 s. The tensile samples were machined using electric wire cutting from the printed blocks. Tensile tests were conducted on three specimens for each condition, and the average values were reported. The tensile tests were conducted at room temperature at a constant strain rate of 10^−3^ s^−1^ using a Shimadzu AG-XD plus (Kyoto, Japan) universal tensile testing machine. The gauge dimensions of the tensile samples were 14 mm × 4 mm × 2 mm. A non-contact Shimadzu video extensometer was employed to measure the tensile strain.

The DIC analysis was performed using MATLAB R2023b software to examine the localized deformation behavior. Figure 1 shows the sample preparation for the DIC test. The tensile sample was first ground using 1000 grit SiC sandpaper (Figure 1b). Then, the tensile sample was painted white (Figure 1c). Then, black paint was sprayed over the white paint (Figure 1d). The black spray was in the shape of micron-sized particles (Figure 1e). The spacings between these particles were monitored during tensile testing using a high-resolution video recorder and were utilized to calculate the localized strains through DIC analysis.

## 3. Results

### 3.1. Microstructures of the LPBF-Fabricated 316L and CuCrZr

**Figure 2:**
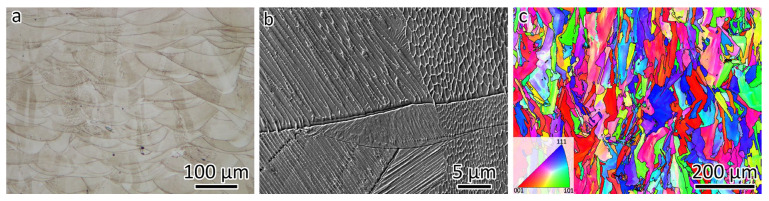
Microstructures of LPBF-fabricated 316L. (**a**) Optical micrograph showing the melt pool morphology. (**b**) SEM image showing the cellular structure. (**c**) EBSD orientation map showing the grain size and morphology.

Figure 2 shows the microstructures of the LPBF-fabricated 316L. The LPBF-fabricated 316L stainless steel exhibits a hierarchical microstructure that includes melt pool boundaries, low-angle grain boundaries (LAGBs, 2–15°), high-angle grain boundaries (HAGBs, >15°), and nanosized cellular structures. The optical micrograph in Figure 2a shows the morphology of the melt pool structures. The overlap between adjacent laser tracks prevents the formation of lack-of-fusion defects. The SEM micrograph in Figure 2b provides a closer view of the nanosized cellular structures, which display both equiaxed and tubular morphologies. The boundary separating the equiaxed and tubular structures is a grain boundary, which can be either a LAGB or a HAGB.

The EBSD orientation map is shown in Figure 2c, which was observed from the same orientation as Figure 2a,b. The typical grain boundary has a misorientation larger than 15°, which is a HAGB. A gradient color change can be observed in the EBSD orientation map (Figure 2c) because of the presence of LAGBs. The black lines in Figure 2c represent the HAGBs. The grain size was measured at 16.4 µm. It can be seen that some grains are larger than 200 µm, which is larger than the layer thickness. This is because of the epitaxial growth of 316L resulting from the layer-by-layer building strategy.

**Figure 3:**
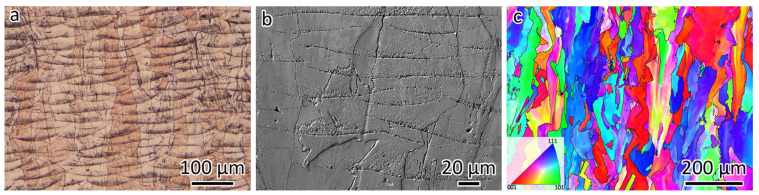
Microstructures of LPBF-fabricated CuCrZr. (**a**) Optical micrograph showing the melt pool morphologies. (**b**) SEM image showing the cellular structure. (**c**) EBSD orientation map showing the grain size and morphologies.

Figure 3 presents the microstructures of LPBF-fabricated CuCrZr. The optical micrograph in Figure 3a reveals the melt pool morphology. Despite employing a higher laser power and a slower scanning speed compared to 316L, the melt pool in CuCrZr remains shallower. This is attributed to the lower laser energy absorption characteristic of Cu-based alloys. A magnified view of the melt pool structure, captured using SEM, is displayed in Figure 3b. Meanwhile, the EBSD orientation map in Figure 3c, observed from the same orientation as Figure 3a,b, indicates a grain size of 20.9 µm. The black lines in Figure 3c represent the HAGBs. Additionally, epitaxial grain growth is evident in the CuCrZr alloy.

### 3.2. Microhardness, Tensile Properties, and Fracture Position of the Bimetal Joint

**Figure 4:**
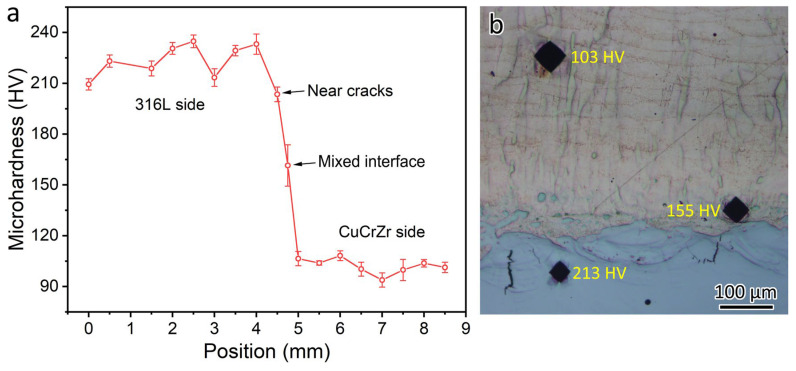
(**a**) Microhardness of the bimetal joint near the interface. (**b**) Locations of the three microhardness indenters near the bimetal joint interface, i.e., 316L-rich zone near a crack (220 HV), immiscible zone (147 HV), and CuCrZr side (103 HV).

The microhardness profile of the 316L/CuCrZr bimetal structure is shown in Figure 4a. The average microhardness was 224 HV for LPBF 316L and 102 HV for LPBF CuCrZr. The microhardness was 161 HV at the mixed interface. Figure 4b shows three microhardness tests conducted near the bimetal joint interface. The locations of the three microhardness indenters are the 316L-rich zone near a crack, the mixed interface, and the CuCrZr side. In the 316L-rich zone, where the cracks are formed, the microhardness of the indenter was 220 HV. The microhardness was 147 HV for the mixed interface and 103 HV for the LPBF-fabricated CuCrZr.

**Figure 5:**
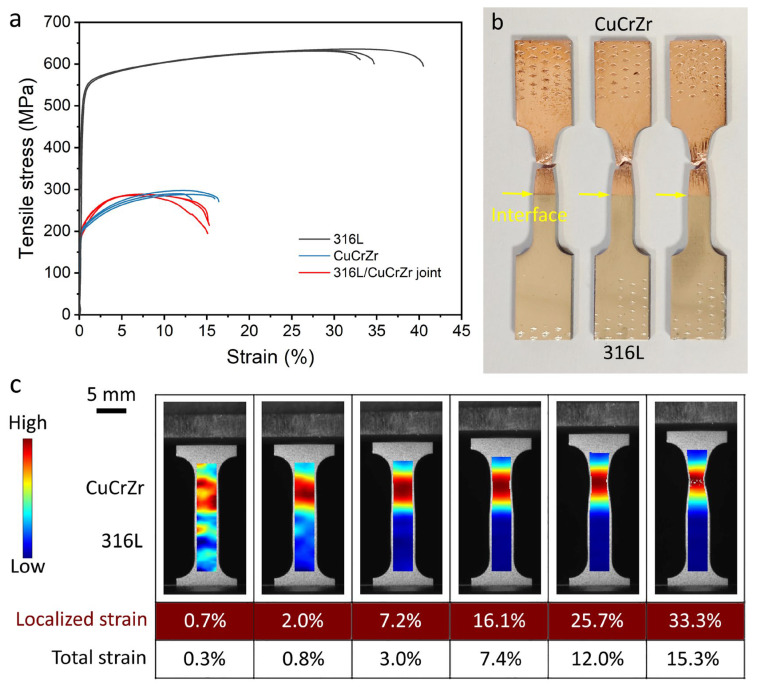
(**a**) Tensile curves of the three 316L/CuCrZr bimetal joints. (**b**) Photo showing the fracture occurring on the CuCrZr side. (**c**) DIC results showing the deformation behavior during tensile test. BD: building direction.

Figure 5a shows the tensile curves obtained from LPBF-fabricated 316L, CuCrZr, and the bimetal joint. The details of the tensile results are shown in Table 3. The yield strength of 316L (507.8 MPa) is higher than that of CuCrZr (201.7 MPa). The 316L/CuCrZr bimetal joints fractured on the CuCrZr side, as shown in Figure 5b, indicating good bonding between 316L and CuCrZr fabricated using the LPBF process without any filler materials. The UTS of CuCrZr and the bimetal joint are similar because the bimetal joint fractured on the CuCrZr side.

The DIC results in Figure 5c show the deformation evolution of 316L/CuCrZr bimetal joints during quasi-static tensile testing. A video showing the DIC results is provided in the Supplementary Information. The red color represents high localized strain, while the blue color represents low localized strain. The values for the highest localized strain and the total strain are also given in Figure 5c. It can be seen that yielding occurs only on the CuCrZr side of the 316L/CuCrZr bimetal joint. The uniform elongation is about 7.4%. Throughout the tensile test, plastic deformation only happens on the CuCrZr side due to its low hardness and strength. This explains why the yield strength of CuCrZr and the bimetal joint is similar. The highest localized strain was 33.3% on the CuCrZr side, while the total strain was 15.3%.

### 3.3. Microstructures at the Interface

**Figure 6:**
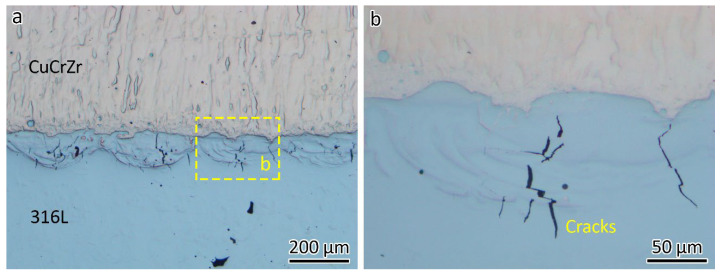
(**a**) Optical images showing the microstructure of the 316L/CuCrZr bimetal structure, (**b)** a close-up.

The optical micrograph in Figure 6a shows the microstructure of the 316L/CuCrZr bimetal structure. Minimal defects were found in the LPBF-fabricated CuCrZr, while a lack of fusion was evident in the LPBF-fabricated 316L, as shown in Figure 6a.

Typically, if a welded joint fractures in the base material, as seen in Figure 5b, it indicates good joint quality. However, microstructural analysis revealed interface cracks, as shown in Figure 6a, with the longest measuring around 150 µm. These cracks generally align with the building direction, as highlighted in the close-up view in Figure 6b.

**Figure 7:**
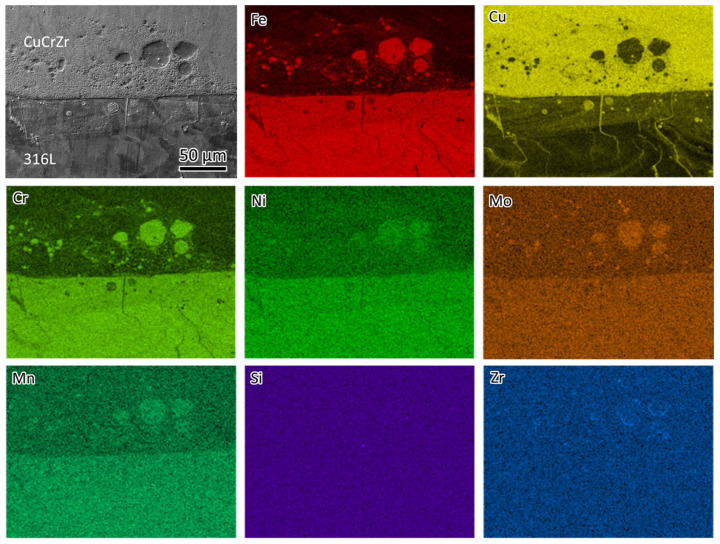
SEM micrograph of the 316L/CuCrZr interface and the EDS mapping showing the element distribution.

Figure 7 shows the elemental distribution at the interface of the bimetal joint. A micron-sized spherical phase was observed on the CuCrZr side, containing Fe, Cr, Ni, Mo, and Mn, indicating that these are 316L phases. A spherical Cu-rich phase was also observed on the 316L side. This is because, when printing the first few layers of CuCrZr, the previous 316L was partially remelted. The presence of spherical 316L and Cu phases was a result of the immiscibility of the Fe-Cu system. Additionally, traces of Cu-rich lines can be observed on the 316L side. The line traces will be studied in a later section.

**Figure 8:**
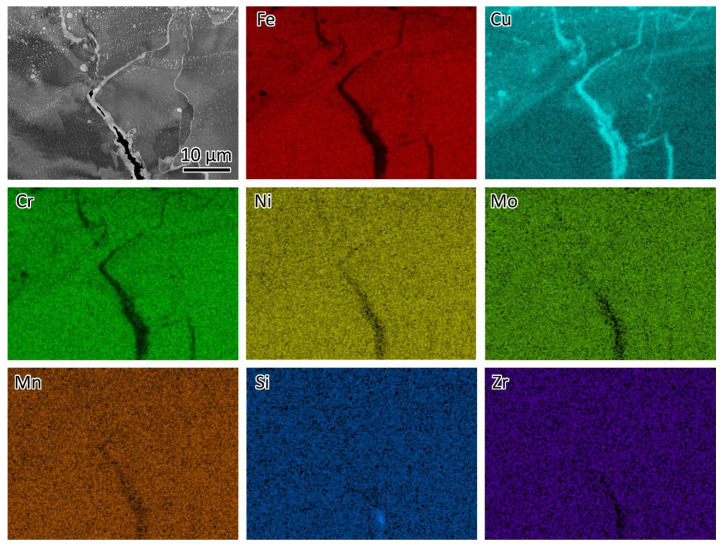
SEM BSE micrograph of a crack and EDS element mapping showing the distribution of the elements near the crack.

**Figure 9:**
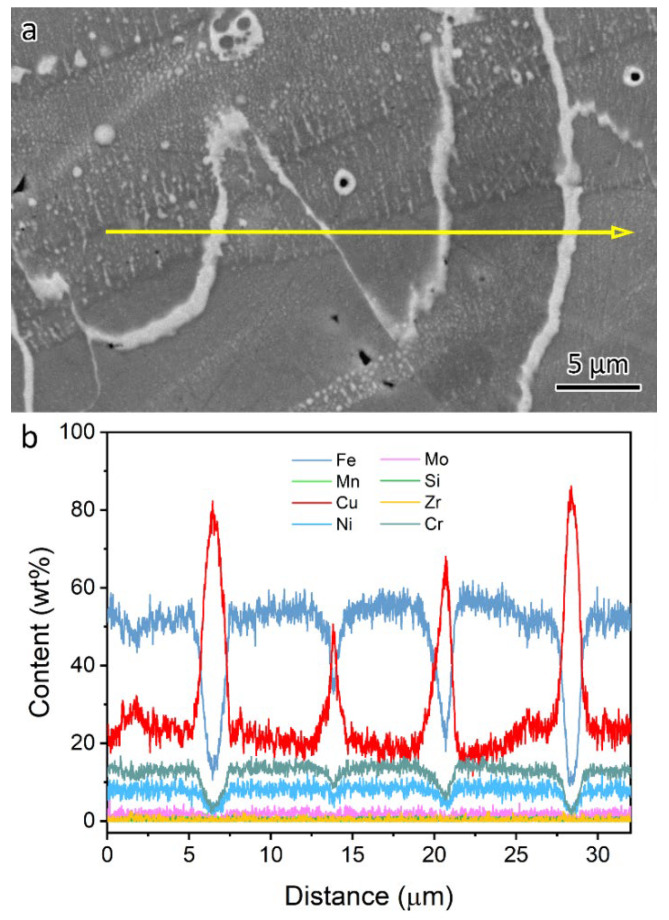
(**a**) EDS line scan showing the (**b**) quantitative chemical composition at the Cu segregations.

In the LPBF-fabricated bimetal structure, 316L stainless steel was initially deposited on a substrate. Subsequently, CuCrZr alloy was printed atop the 316L. As a result, the initial layers of the CuCrZr contained a mixture of 316L and CuCrZr. Cracks were observed in these mixed layers, as shown in Figure 6 and Figure 8. Figure 8 presents an SEM backscatter electron (BSE) micrograph of a crack, accompanied by EDS element mapping illustrating the elemental distribution near the crack. Significant Cu segregation was observed in these regions. The line traces in Figure 7 indicate the Cu segregations. Figure 9 provides an EDS linescan, showing the quantitative chemical composition at the Cu segregations, with Cu content ranging from 51 wt% to 86 wt%.

The melting point is 1430 °C for 316L and 1085 °C for pure copper. Cu segregation occurred along the cracks due to the immiscibility of the Cu–Fe system. During melting, the mixture of 316L and Cu alloy formed a molten phase. As the material solidified, 316L solidified first because of its higher melting point, rejecting Cu solutes to the liquid/solid interface, where they segregated at the grain boundaries. In the temperature range between 1430 °C and 1085 °C, Cu remained in a liquid state. Upon further cooling and solidification, Cu solidified with volume shrinkage, contributing to crack formation in the Cu segregation zones. During solidification, thermal stress also contributed to the formation of cracks. Based on this discussion, the cracks are classified as hot cracks [17], which occur during solidification as the low-melting elements, e.g., Cu, partition into the grain boundaries of 316L.

Previous research on laser welding of steel/Cu dissimilar joints suggests that the cracks are highly sensitive to the content of Cu [18]. A higher content of Cu promotes the formation of cracks, as shown in Figure 8, whereas no cracks were observed along the thinner Cu track. The formation of cracks can be avoided by limiting the content of Cu infiltration into steels [19]. Adding Cu to 316L slows grain growth during the annealing process after rolling [20] and can also enhance the antibacterial properties of 316L [21]. This study aims to inspire further research into strategies for mitigating crack formation. Introducing an interlayer between 316L and CuCrZr could be a potential solution. Li et al. [16] used Inconel 718 as an interlayer, successfully preventing crack formation.

**Figure 10:**
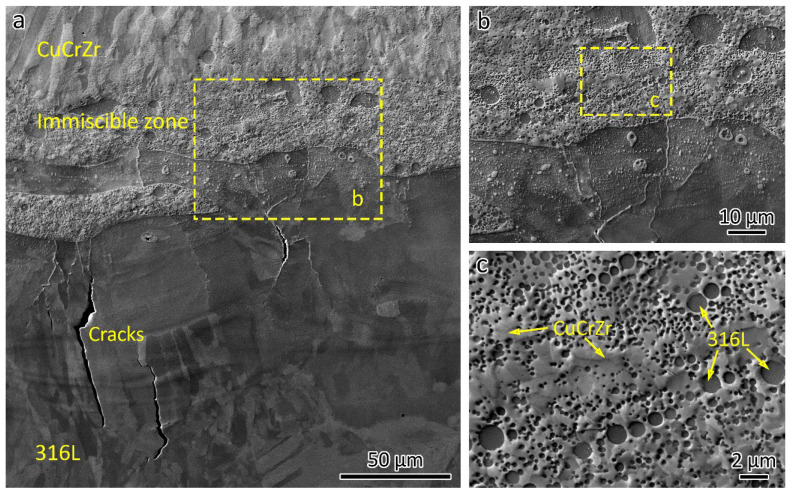
SEM micrographs showing the microstructures near the bimetal interface: (**a**) an overview showing the cracks at the 316L-rich zone and interface, (**b**) a close-up showing the microstructures 316L-rich zone and CuCrZr-rich zone, (**c**) the immiscible zone.

The SEM micrographs in Figure 10 show the microstructures near the bimetal interface. Figure 10a provides an overview, highlighting the cracks within the 316L-rich zone, consistent with observations from Figure 6. A closer view of the microstructures in the 316L-rich and CuCrZr-rich zones is shown in Figure 10b, while Figure 10c illustrates the immiscible zone, with arrows indicating the 316L and CuCrZr regions. Additionally, the size of Fe-rich spheroids in the microstructure of immiscible Cu–Fe alloys increases with greater undercooling [22].

**Figure 11:**
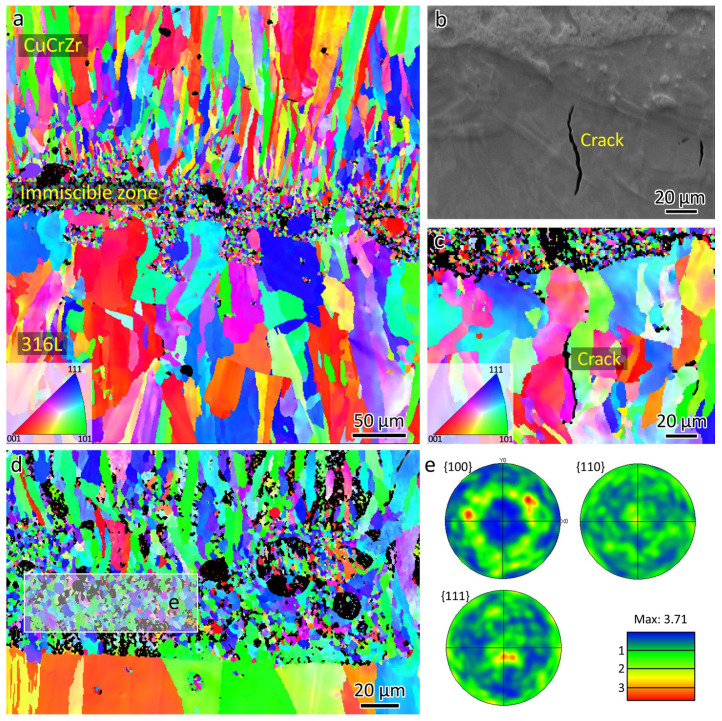
(**a**) EBSD orientation map showing the microstructures near the bimetal interface, (**b**) SEM micrograph showing the crack at the interface, (**c**) EBSD orientation map showing crack at the grain boundary, (**d**) EBSD orientation map showing the microstructure at the immiscible zone, and (**e**) pole figure showing the preferential <111> orientation.

Figure 11a presents the EBSD analysis of the microstructures near the bimetal interface, revealing a columnar grain morphology in both LPBF-fabricated 316L and CuCrZr, with smaller grains at the interface due to Fe-Cu immiscibility.

Figure 11b shows the SEM micrograph of a crack obtained from the 316L-rich zone. Figure 11c presents the corresponding EBSD orientation map of the area in Figure 11b. It can be seen that the crack is formed along the HAGBs.

The grains in the mixed zone exhibit a weak preferential <111> orientation, as confirmed by the EBSD pole figure analysis shown in Figure 11d,e, where the maximum texture index is close to 3.71. Similar results have been found by Li et al. [16]. It has been proven that preferential orientation can be retarded through grain refinement [23]. An orientation relationship is typically observed between precipitates and the matrix [24] or during phase transformations [25]. However, in the Fe-Cu system, which is immiscible, the submicron-sized 316L within the CuCrZr matrix (Figure 10c) is not a precipitate. Instead, it is randomly oriented within the CuCrZr matrix. Therefore, no preferential orientation is present.

The grains in the immiscible zone are small, measuring approximately 5 µm. In contrast, LPBF-fabricated CuCrZr has a grain size of 20.9 µm. There could be two reasons for the smaller grain size in the immiscible zone.

First, the presence of submicron-sized 316L spherical phases may act as heterogeneous nucleation sites for CuCrZr grains (Figure 10c). Before printing CuCrZr, the 316L powder must be removed, and the CuCrZr powder loaded. The building chamber then needs to be vacuumed. After this process, the LPBF-fabricated 316L has already cooled to room temperature, which means that CuCrZr is printed on a cold substrate. This thermal condition could be the second reason for the smaller grain size in the initial layers.

**Figure 12:**
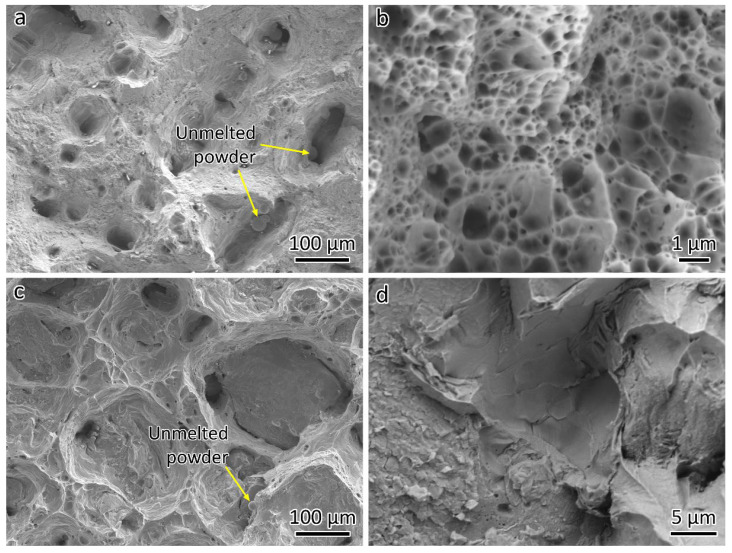
SEM micrographs showing the fractographs of (**a**,**b**) 316L and (**c**,**d**) CuCrZr.

Figure 12a presents the fractographs of the LPBF-fabricated 316L, where un-melted powder is visible, indicating the presence of lack-of-fusion defects. Un-melted or partially melted powders have frequently been observed in lack-of-fusion defects [26]. Typically, LPBF-fabricated 316L with high relative density exhibits good ductility, such as 59% [27] and 62% [23]. The low ductility observed in this study is attributed to the presence of lack-of-fusion defects (Figure 6a). A close-up view in Figure 12b reveals nanosized dimples.

Figure 12c shows the fracture surface that was taken from the 316L-CuCrZr joint sample. Since the joint fractured on the CuCrZr side, the fractographs are actually for the CuCrZr sample. Due to the high relative density of CuCrZr, fewer un-melted powder particles are observed, as seen in Figure 12c. A close-up view of the fractography is provided in Figure 12d.

## 4. Conclusions

- Tensile fracture occurring in the base material is not a definitive indicator of joint quality. A comprehensive microstructural analysis of the weld interface is crucial to assess the integrity of dissimilar material bonding;
- The grain size is 16.4 µm for LPBF-fabricated 316L and 20.9 µm for LPBF-fabricated CuCrZr. The grain size at the 316L/CuCrZr interface is approximately 5 µm. The primary reason for this small grain size is the formation of submicron-sized spherical 316L particles, which act as heterogeneous nucleation sites for CuCrZr due to the immiscibility of the Fe-Cu system;
- Cracks were observed at the 316L/CuCrZr interface, primarily near the 316L side, despite the tensile fracture occurring in the base material. EBSD analysis confirmed that the crack forms along the high-angle grain boundaries of 316L. The underlying mechanism is the immiscibility of the Fe-Cu system, which causes Cu to segregate at the grain boundaries;
- The LPBF-fabricated 316L/CuCrZr bimetal joint exhibits a yield strength of 203.0 MPa, a UTS of 287.5 MPa, and an elongation of 15.3%. The strength of the bimetal joint is similar to that of CuCrZr because the joint deforms and fractures only on the CuCrZr side during the tensile test. The bimetal structure fractured on the CuCrZr side due to its relatively lower strength compared to 316L (yield strength: 507.8 MPa, UTS: 633.3 MPa).

## Figures and Tables

**Table 1:** Nominal chemical compositions of 316L and CuCrZr powders.

**316L**	**Fe**	**Cr**	**Ni**	**Mo**	**Mn**	**Si**	**C**
	Balance	16–18	10–14	2–3	<2	<0.75	<0.03
CuCrZr	**Cu**	**Cr**	**Zr**				
	Balance	0.5–1.2	0.05–0.25				

**Table 2:** LPBF parameters for the printing of 316L and CuCrZr.

Alloy	Laser Power (W)	Scan Speed (mm/s)	Layer Thickness (µm)	Hatching Spacing (µm)
316L	220	1100	30	90
CuCrZr	375	700	30	100

**Table 3:** Tensile test results obtained using different samples.

Sample	Yield Strength (MPa)	UTS (MPa)	Elongation (%)
316L	507.8 ± 12.8	633.3 ± 2.6	36.1 ± 4.0
CuCrZr	201.7 ± 4.1	291.8 ± 5.0	15.2 ± 1.7
Joint	203.0 ± 9.3	287.5 ± 1.5	15.3 ± 0.1

## Data Availability

The original contributions presented in the study are included in the article, further inquiries can be directed to the corresponding author.

## Supplementary Materials

The following supporting information can be downloaded at https://www.mdpi.com/article/10.3390/ma18071434/s1, Video S1: LPBF 316L_CuCrZr DIC.
